# Automated system for classification of COVID-19 infection from lung CT images based on machine learning and deep learning techniques

**DOI:** 10.1038/s41598-022-20804-5

**Published:** 2022-10-18

**Authors:** Bhargavee Guhan, Laila Almutairi, S. Sowmiya, U. Snekhalatha, T. Rajalakshmi, Shabnam Mohamed Aslam

**Affiliations:** 1 Department of Biomedical Engineering, College of Engineering and Technology, SRM Institute of Science and Technology, Kattankulathur, Tamil Nadu 603203 India; 2 Department of Computer Engineering, College of Computer and Information Sciences, Majmaah University, Al Majmaah, 11952 Saudi Arabia; 3 Department of Electronics and Communication Engineering, College of Engineering and Technology, SRM Institute of Science and Technology, Kattankulathur, India; 4 Department of Information Technology, College of Computer and Information Sciences, Majmaah University, Al Majmaah, 11952 Saudi Arabia

**Keywords:** Diseases, Health care, Engineering

## Abstract

The objectives of our proposed study were as follows: First objective is to segment the CT images using a k-means clustering algorithm for extracting the region of interest and to extract textural features using gray level co-occurrence matrix (GLCM). Second objective is to implement machine learning classifiers such as Naïve bayes, bagging and Reptree to classify the images into two image classes namely COVID and non-COVID and to compare the performance of the three pre-trained CNN models such as AlexNet, ResNet50 and SqueezeNet with that of the proposed machine learning classifiers. Our dataset consists of 100 COVID and non-COVID images which are pre-processed and segmented with our proposed algorithm. Following the feature extraction process, three machine learning classifiers (Naive Bayes, Bagging, and REPTree) were used to classify the normal and covid patients. We had implemented the three pre-trained CNN models such as AlexNet, ResNet50 and SqueezeNet for comparing their performance with machine learning classifiers. In machine learning, the Naive Bayes classifier achieved the highest accuracy of 97%, whereas the ResNet50 CNN model attained the highest accuracy of 99%. Hence the deep learning networks outperformed well compared to the machine learning techniques in the classification of Covid-19 images.

## Introduction

In December 2019, the World Health Organization (WHO) declared that the Coronavirus outbreak a global pandemic. The causative agent behind the pandemic is the SARS-Cov-2 virus, a class of Coronavirus responsible for attacking the respiratory system of the infected individuals. The onslaught of the pandemic has reached an outbreak of 150 million cases with infection (as on April 2021) and resulted in a death rate of about 20%^1^. Common symptoms of infection include fever, cold, sore throat, cough, shortness of breath, and fatigue. Lung complications, multi-organ dysfunction, and critical illness are found to occur in severe cases. Studies have also found a link between COVID-19 infection and complications associated with the myocardial system^2^. Infected individuals can either shows symptoms and there are cases without any visible symptoms but still be a carrier of the virus.

Tests for the detection of COVID-19 may be classified as serological, nucleic acid, antigen, and ancillary tests, all of which play distinguished roles in hospitals and healthcare^3^. The most commonly used test is the Reverse Transcription Polymerase Chain Reaction (RT-PCR). RT-PCR is often regarded as the gold standard method for COVID-19 detection due to its correct prediction rate, high sensitivity and specificity^4^. An important issue associated with real-time RT-PCR tests is the false-positive and false-negative results. There have been cases where ‘suspected’ COVID-19 patients with infections diagnosed in their CT scans were not interpreted correctly by the real-time RT-PCR tests^5^. The primary reason behind this is speculated to be the rapid mutations and genetic diversity of the Coronavirus^6^.

Ever since the onset of the pandemic, there has been much research into the clinical and radiological manifestations of the COVID-19 virus. The primarily targeted region of interest of COVID-19 is the respiratory system which is evident from the chest imaging (radiography and computed tomography) has shown significant results. Manifestation of COVID-19 pneumonia in chest imaging occurs in bilateral peripheral ground-glass opacities and lower lung distribution. The opacities sometimes have areas of consolidation and have a node-like or mass-like appearance. In clinical practice, patients with predominate ground-glass opacities in the upper lobes are subjected to COVID-19^7^. Computed tomography has shown to have greater sensitivity than chest x-ray imaging in detecting COVID-19 pneumonia^8^.

An increasing amount of research is being conducted in artificial intelligence and deep learning to aid the diagnosis of COVID-19^9^. Jain et al.^10^ implemented deep learning-based detection of COVID-19 from chest radiographs. They compared the performance of pre-trained models Inception V3, Xception, and ResNet in classifying radiographs of normal subjects and COVID-19 patients. Out of the three models, the Xception model attained the highest accuracy of 97%. Apostolopoulos et al.^11^ utilized transfer learning with CNN networks to classify chest x-ray (CXR) images of common cold, COVID-19, and healthy controls. High accuracy of 97% was achieved using VGG-19 architecture and transfer learning technique. Shah et al.^12^ employed deep learning methods to differentiate COVID and non-COVID CT images. DenseNet, VGG-19, ResNet, and Inception models were used, out of which VGG-19 achieved the highest accuracy of 94.5%. Singh et al.^13^ classified chest CT images as COVID-19 positive and negative cases using multi objective differential equation (MODE) based CNN and achieved high accuracy of 92%. Narin et al.^14^ compared the performance of five pre-trained neural network models in detecting COVID-19 infection from chest X-ray images. ResNet50 achieved the highest accuracy of 96.1% using fivefold cross-validation out of the five models.

Raajan et al.^15^ developed an accurate, high speed and more sensitive CT scan approach towards Covid-19 diagnosis. The authors used ResNet50 architecture to train the image data set. It was observed that the infected individuals were identified correctly by comparing the testing dataset with the training data set. ResNet 50 has predicted Covid–19 with an accuracy and specificity of about 95.09 and 81.89% respectively. The accuracy of ResNet50 is high compared to other networks such as Alexnet, ZFNet, GoogLe Net and VGG Net. Abdul Kareem et al.^16^ developed a diagnostic system for Covid-19 using deep learning techniques such as CNN, autoencoder and deep neural network. They obtained CT image dataset from free publicly available radiology resources. They segmented the lung CT image using histogram thresholding method and morphological dilation operation. They constructed the CNN architecture using convolution layer with varying filter size and used softmax layer for the classification of covid-19 images. The accuracy of CNN and DNN model obtained is 88.3 and 86.23% respectively. The authors segmented the lung CT image but the ground class opacity in covid infected regions is not segmented. Salama et al.^17^ introduced a generalized framework to segment and classify the lung CT images of covid -19 patients. They used transfer learning based ResNet 50 and VGG16 network for the classification covid-19 and healthy images. The authors employed U-Net segmentation method for segmentation of lung CT images. The authors implemented the proposed work with the existing Kaggle datasets. The combination of U-Net with VGG16 and ResNet 50 achieved better performance accuracy of 98.98%. They used U-net for segmentation of the lung CT images, but the ground class opacities due to the inflammation is not segmented well. In the existing literature, most of the works focussed only on detection of Covid-19 with deep learning techniques, few studies focussed on segmentation of the Covid-19 region and classification using machine learning techniques. But our proposed study concentrates on segmentation of ground class opacites in covid-19 infected regions of lung CT images, followed by feature extraction process and further classified using machine learning classifiers. In addition to that, automated features are extracted using the pre-trained convolution neural networks based on the transfer learning approach. The performance of deep learning techniques are compared with that of the machine learning techniques.

The proposed study involves segmentation of lung CT images using clustering-based approach like k-means algorithm and extracting the abnormal ground class opacities in Covid-19 images. Then feature extraction was performed using GLCM algorithm and extracted features are fed into the machine learning classifiers such as Naïve Bayes, Bagging, and REP Tree for the classification of Covid and Non-covid images. Furthermore, after data augmentation, the images are fed into the deep learning pre-trained CNN such as modified Alex Net, ResNet50, and Squeeze Net based on transfer learning approach. Finally, the performance of transfer learning based pre-trained CNN models were compared with that of the machine learning classifiers. The proposed approach gained better classification accuracy with less computational complexity and execution time compared to existing literatures. Moreover, instead of analysing data obtained from publicly available datasets, real patient data were analysed.

The aim and objectives of our study can be summarized as follows:

1. To detect the ground class opacities in Covid-19 images by applying segmentation techniques like k-means clustering algorithm in lung CT images.
2. To extract the features from the segmented image regions using GLCM method followed by implementation of machine learning algorithms for the classification of the images into COVID and non-COVID image classes.
3. To implement the pre-trained CNN models for classifying images into the two classes and to compare its performance with the machine learning classifiers.

## Results

### Segmentation

**Figure 1:**
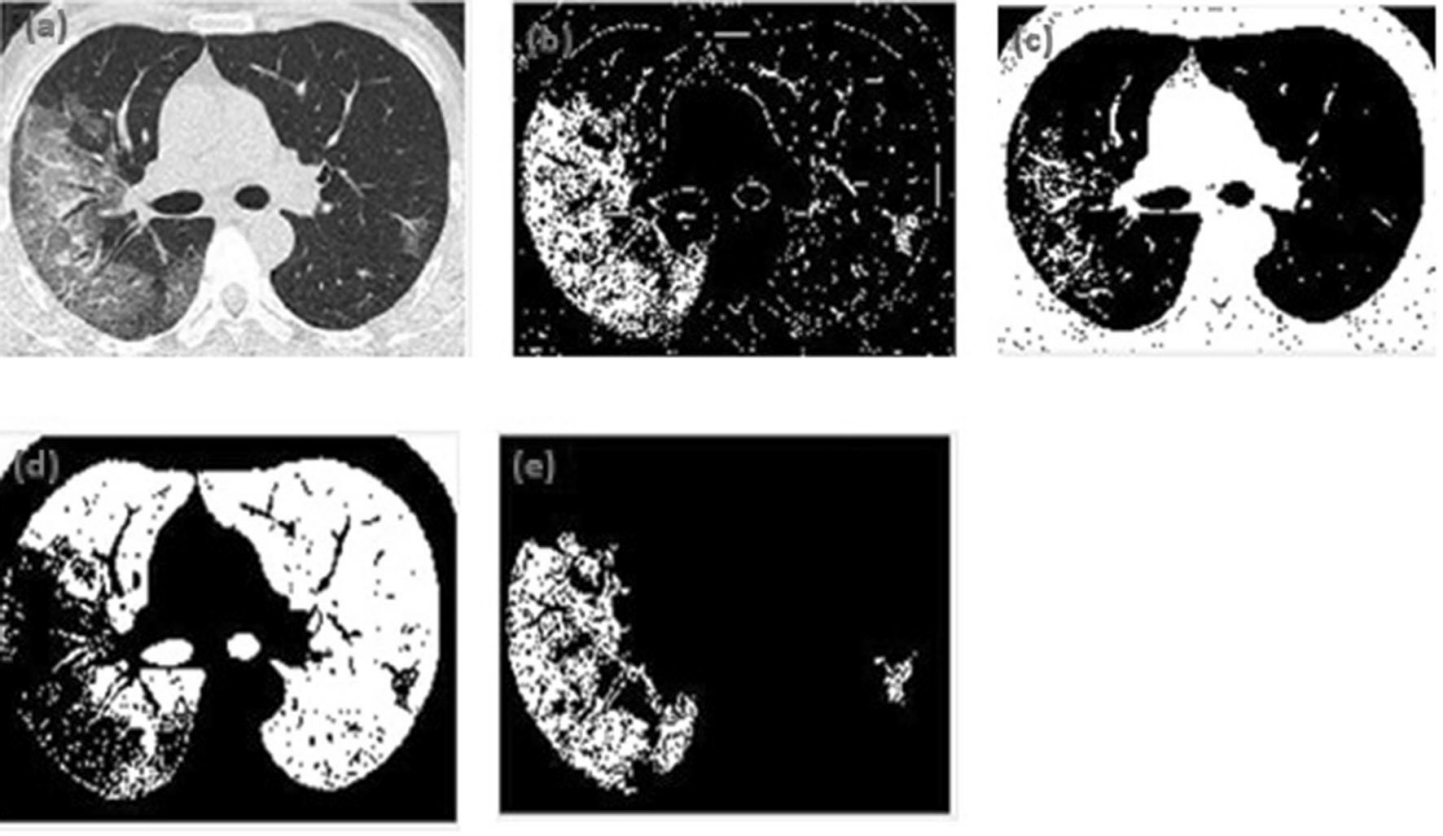
*k*-means segmentation of COVID CT image (**a**) original CT image of COVID-19 patient, (**b**) cluster depicting the region of interest, (**c**) cluster that detects several edges in the image, including the lungs, vessels, and some of the ground glass opacities, (**d**) cluster depicting uninfected regions of the lungs, (**e**) final *k*-means image highlighting lung infection such as ground-glass opacities.

**Figure 2:**
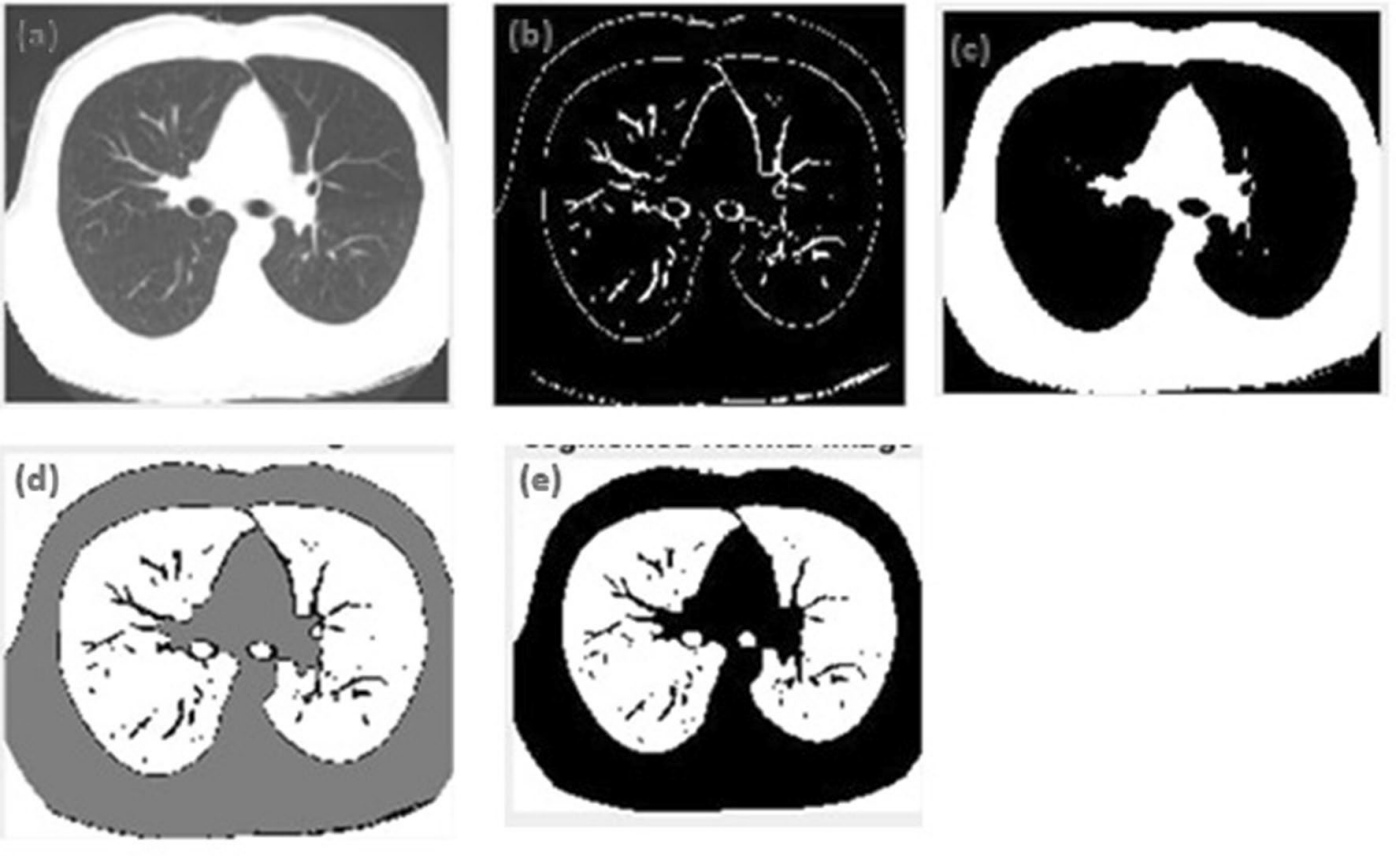
*k*-means Segmentation of non-COVID CT image (**a**) original non-COVID CT image, (**b**) cluster that depicts the edges detected in the image, (**c**) cluster depicting the surrounding lung region, (**d**) cluster depicting the region of interest, (**e**) depicts final k-means segmented image.

The raw CT images from the dataset were pre-processed, and segmented using *k*-means clustering algorithm. The number of clusters in the algorithm was set to 3. Cluster 1 depicts the desired ROI. The segmentation results for both the image classes have been displayed in Figs. 1 and 2. Figure 1 represents the segmented CT image of an individual infected with COVID-19. Figure 2 shows the segmented CT image of the non-COVID subject. It is observed that the first cluster depicted the desired region of interest from the COVID and non-COVID CT images.

Figure 1 (a) represents the original CT image of COVID-19 patient, (b) indicates the cluster depicting the region of interest, (c) shows the cluster that detects several edges in the image, including the lungs, vessels, and some of the ground glass opacities, (d) represents the cluster depicting uninfected regions of the lungs, (e) depicts the final *k*-means image highlighting lung infection such as ground-glass opacities due to COVID-19.

Figure 2 (a) represents the original non-COVID CT image, (b) represents the cluster that depicts the edges detected in the image, (c) shows the cluster depicting the surrounding lung region, (d) indicates the cluster depicting the region of interest, (e) depicts final k-means segmented image.

### Feature extraction

Hand-crafted features were extracted from the segmented images before the classification process. The GLCM algorithm was employed to extract six textural and statistical features (Table 1).

**Table 1:** GLCM Feature Extraction.

Feature	COVID	Non-COVID
Mean	0.15 ± 0.13	0.21 ± 0.18
Contrast	0.08 ± 0.03	0.06 ± 0.04
Energy	0.69 ± 0.15	0.67 ± 0.23
Homogeneity	0.95 ± 0.01	0.96 ± 0.02
Correlation	0.59 ± 0.13	0.59 ± 0.24
Entropy	0.53 ± 0.24	0.57 ± 0.37

It is observed that the contrast value of the COVID class was found to be greater than that of the non-COVID class by 9%. Due to the presence of ground-glass opacities in the lung which is translucent/opaque and that could contribute towards high contrast compared to the other regions. The energy of COVID images is more elevated than non-COVID images by 7%. The high energy of COVID class images indicates higher pixel intensity values. Homogeneity of the non-COVID class was found to be 2% greater than the COVID class. The entropy was found to be 22% higher for the non-COVID image with respect to COVID image. Entropy and homogeneity measure randomness and uniformity in an image, respectively. These parameters indicate that the CT manifestation of COVID-19 pneumonia contributes towards decrease in the image randomness. 15% correlation was observed between the non-COVID and COVID images. The mean of the non-COVID images was also more significant than the images belonging to the COVID class. The mean value was 39% lesser in COVID images than in non-COVID images. It is inferred that the presence of ground glass opacities, consolidation, and other signs of infection in the CT images leads to a decrease in the overall average contribution of pixels towards the mean intensity value.

### Machine learning classification

This study involves three machine learning classifiers, namely Naive Bayes, Bagging and REPTree for the classification of Covid-19 and Normal images. The classifier’s performance was analyzed and evaluated to find which classifier provides higher accuracy. A confusion matrix compared the actual values with the values predicted by the model. Using the parameters true positive, false positive, false negative, and true negative, performance metrics such as accuracy, precision, and sensitivity was calculated. Further, the receiver operator characteristics curve (ROC), a plot of true positive and false positive rates was generated. The area under curve (AUC) was also analysed for the proposed classifiers.

It was found that the highest accuracy was achieved by the Naive Bayes classifier, attaining a value of 97%. Accuracy is an indicator of the number of correct predictions made by the classifiers. All the three classifiers such as Naïve Bayes, Bagging, and REPTree classifier, achieved an accuracy of 97, 96 and 93%, respectively. Both Naïve Bayes and Bagging attained the highest precision of 96%. The REPTree attained a precision of 93%, which is also a good level of performance. The sensitivity value of Naïve Bayes classifier was found to be 97% in comparison with that of Bagging (96%), and REPTree (93%). Table 2 depicts the confusion matrix of the proposed classifiers.

**Table 2:** Confusion Matrix for Naïve Bayes, Bagging and REPTree.

n = 100	Predicted positive (COVID)	Predicted negative (non-COVID)	%
**Naïve Bayes classifier**			
Actual positive (COVID)	(TP) 48	(FN) 2	Sensitivity 97
Actual negative (non-COVID)	(FP) 1	(TN) 49	Precision 96
			Accuracy 97
**Bagging classifier**			
n = 100	Predicted positive (COVID)	Predicted negative (non-COVID)	%
Actual positive (COVID)	(TP) 48	(FN) 2	Sensitivity 96
Actual negative (non-COVID)	(FP) 2	(TN) 48	Precision 96
			Accuracy 96
**REPTree classifier**			
n = 100	Predicted positive (COVID)	Predicted negative (non-COVID)	%
Actual positive (COVID)	(TP) 46	(FN) 4	Sensitivity 93
Actual negative (non-COVID)	(FP) 3	(TN) 47	Precision 92
			Accuracy 93

*TP* Number of COVID images correctly classified as COVID, *FP* Number of non-COVID images incorrectly classified as COVID, *TN* Number of correctly classified non-COVID images and *FN* Number of COVID images incorrectly classified as non-COVID.

**Figure 3:**
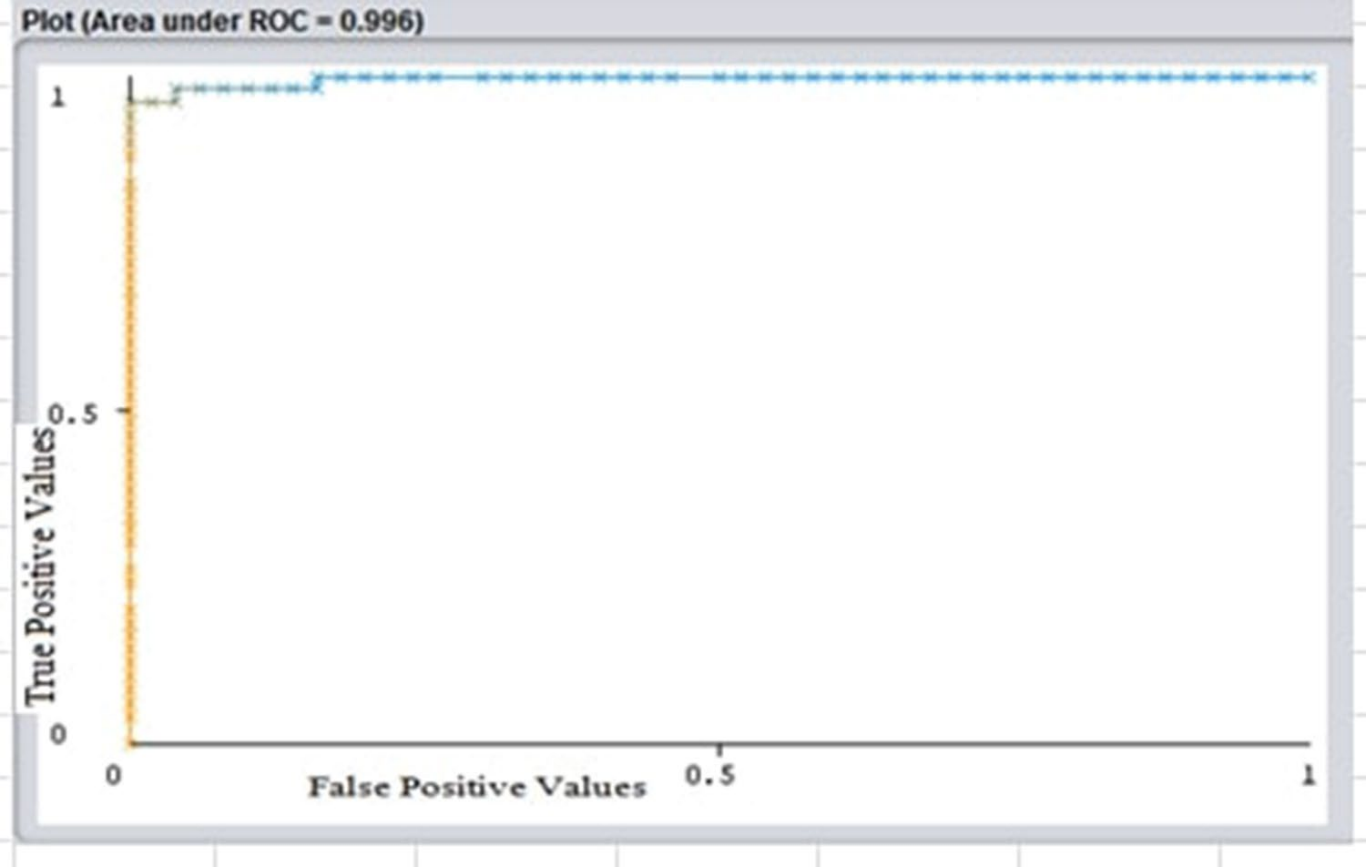
ROC curve of Naïve Bayes classifier.

The ROC curve of the Naive Bayes classifier is depicted in Fig. 3. The AUC value of the curve is a valuable indicator of the classifier's performance. Naive Bayes and Bagging classifier both achieved an AUC value of 0.99. REPTree classifier achieved an AUC value of 0.98.

### Deep learning classification

**Figure 4:**
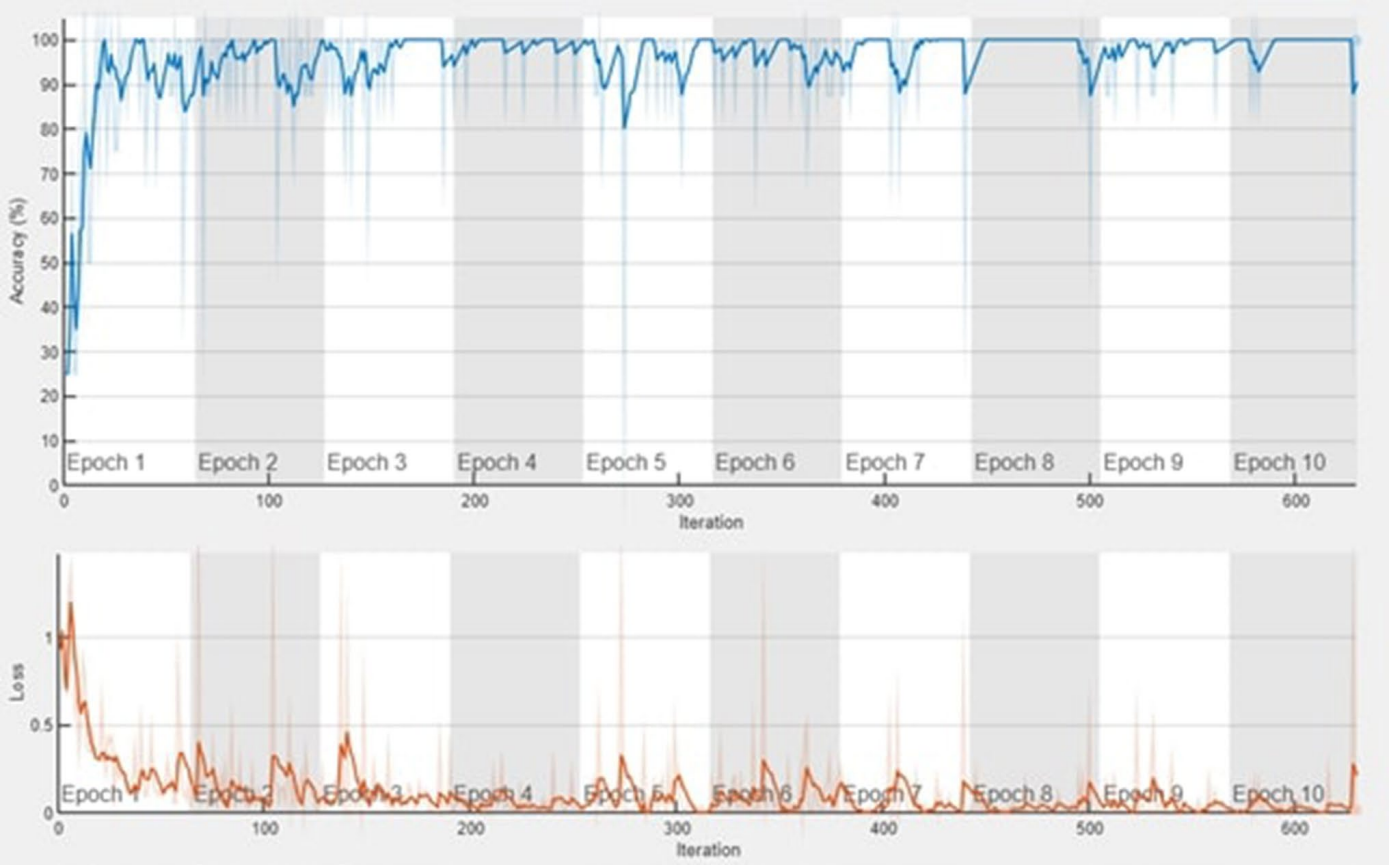
Training accuracy and loss of ResNet50.

**Figure 5:**
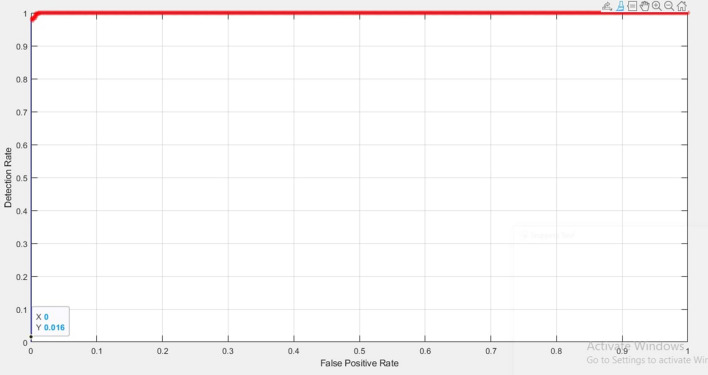
ROC curve of ResNet50.

In this study, three pre-trained CNN models such as Alex Net, ResNet50 and SqueezeNet were trained using transfer-learning approach. At the end of tenth epoch, the training error has descended to a minimum value, whereas the accuracy of the Alex Net model was found to be 96% and the AUC value from the ROC graph was found to be 0.99. In ResNet50 model, at the end of tenth epoch, the error has been reduced to a minimum value resulting in improved training accuracy. The ResNet50 model attained an accuracy of 99.1% and the AUC value was found to be 0.99. Figures 4 and 5 depict the training accuracy and loss and ROC curve, respectively obtained for the ResNet50 architecture.

Squeeze Net architecture attained an accuracy of 94%. The AUC value from the ROC curve was observed to be 0.95. Table 3 displays the confusion matrix along with the accuracy, precision and sensitivity of the three CNN architectures. It can be observed that AlexNet architecture obtained the sensitivity value of 97% and precision of 96%. ResNet50 had a sensitivity of 98% and precision of 99%. Finally, Squeeze Net architecture had the highest sensitivity of 100%, but the precision was only 89%, which was low compared to the other CNN models. Overall, ResNet50 has the best accuracy, good sensitivity and precision for the classification process. The AUC value was also found to be high for ResNet50 (0.99). Although AlexNet attained the same AUC value, the accuracy was low in comparison with ResNet50.

**Table 3:** Confusion matrix for AlexNet, ResNet50 and SqueezeNet.

n = 1000	Predicted positive (COVID)	Predicted negative (non-COVID)	%
**AlexNet**			
Actual positive (COVID)	(TP) 486	(FN) 14	Sensitivity 97
Actual negative (non-COVID)	(FP) 19	(TN) 491	Precision 96
			Accuracy 96.7
**ResNet50**			
n = 1000	Predicted positive (COVID)	Predicted negative (non-COVID)	%
Actual positive (COVID)	(TP) 494	(FN) 6	Sensitivity 98
Actual negative (non-COVID)	(FP) 3	(TN) 497	Precision 99
			Accuracy 99.1
**SqueezeNet**			
n = 1000	Predicted positive (COVID)	Predicted negative (non-COVID)	%
Actual positive (COVID)	(TP) 500	(FN) 0	Sensitivity 100
Actual negative (non-COVID)	(FP) 56	(TN) 444	Precision 89
			Accuracy 94.4

*TP* Number of COVID images classified correctly, *FP* Number of non-COVID images incorrectly classified as COVID, *TN* Number of correctly classified non-COVID images and *FN* Number of COVID images incorrectly classified as non-COVID.

Table 4 summarizes the performance metrics of the machine learning classifiers and the deep learning CNN architectures trained based on transfer learning. Based on the proposed method, it was found that the Naive Bayes classifier attained the best accuracy and would be most suitable for the classification process. It performed well in terms of accuracy, precision, and sensitivity and acquired a high AUC value of 0.99. Based on the three pre-trained CNN model, ResNet50 model outperformed in comparison with other model for the classification of COVID and non-COVID images.

**Table 4:** Summary of performance metrics.

Approach		Accuracy %	Sensitivity%	Precision%	AUC
Proposed method	**Naïve Bayes**	**97**	**97**	**96**	**0.99**
	Bagging	96	96	96	0.99
	REPTree	93	93	92	0.98
CNN transfer learning	AlexNet	96.7	96	97	0.99
	**ResNet50**	**99.1**	**99**	**98**	**0.99**
	SqueezeNet	94.4	89	100	0.95

Significant values are in [bold].

## Discussions

The proposed study involves a novel segmentation and classification using machine and deep learning methods for classifying lung CT images into COVID and non-COVID images. Sahu et al.^18^ performed lung nodules growth measurement and prediction using *k*-means segmentation method with thresholding and morphological operation. They achieved the accuracy of 97.52% and attained the jaccard similarity index value as 0.968. Rathod et al.^19^ developed an automated method for segmentation of covid-19 images using k-means clustering algorithm and used CNN for the classification of covid-19 and non-covid -19 images. They used 4 clusters with black, white, dark gray and light gray colors for segmentation. They trained their proposed CNN model with segmented images and obtained better accuracy of 93.9% compared to pre-trained models ResNet 50, VGG19 and Inception V3. Amyar et al.^20^ implemented multi-task deep learning pre-trained model for the segmentation of lesion and detection of covid-19 images. The authors obtained three different datasets from the hospitals and publicly available dataset. They used encoder and decoder based on U-net architecture and multi-layer perceptron for reconstruction of images and segmentation and classification of injected lesions. They attained the dice-co-efficient as 0.88 for segmentation and yielded good accuracy of 97% for the classification of Covid -19 images.

The proposed study used the k-means clustering algorithm with 3 clusters to segment the infected COVID region in which ground class opacities are segmented. The handcrafted features are extracted from the segmented output images using GLCM algorithm. Then these handcrafted features are fed into the machine learning classifiers such as Naïve bayes, Bagging and Rep tree and yielded the classification accuracy of 97, 96 and 93% respectively. The deep learning networks such as Alex net, ResNet 50 and Squeeze net achieved an accuracy of 96, 99 and 94% respectively.

Zhang et al.^21^ developed the bagging dynamic deep learning network (BDLLN) for the detection of covid-19 based on the symptoms in chest X-ray images. They constructed the B-DDLN network using five convolution layers followed by pooling layers for automated feature extraction from the chest X-ray images. Then the extracted features are fed into the N number of dynamic learning network. Based on the majority voting rule, final diagnosis of the Covid-19 is determined. They achieved 98.8% accuracy using bagging dynamic learning networks for the detection of Covid-19. They compared the BDDLN with the accuracy obtained from the machine learning classifiers such as Naïve bayes (90%), Linear SVM (96.7%) and logistic regression (90%). They predicted that BDDLN outperforms the machine learning classifiers in the classification of Covid-19 and normal images.

Minaee et al.^22^ performed automated detection of Covid-19 from chest X-ray images. The authors collected the data from publicly available dataset of about 5000 chest X-ray images. They used pre- trained models used as ResNet18, ResNet 50, squeezenet and Dense Net161 for the detection of Covid-19 from the chest X-ray image dataset. They provided the heat maps of infected region of Covid-19 using deep visualization techniques. Among the four pre-trained models used, ResNet50 and squeeze net produced an average precision value as 0.899 and 0.897 respectively. The best performing model such as squeeze net and ResNet 18 obtained a better sensitivity and specificity as 98 and 92% respectively. They obtained the accuracy of 89.5 and 92.3% for ResNet 18 model and squeeze net model respectively. Nayak et al.^23^ conducted the study on early detection of covid-19 using chest X-ray images. The authors collected the data of chest X-ray images form the publicly available dataset repository. They used eight pre-trained deep CNN models such as VGG-16, Inception-V3, ResNet 50 and squeeze net for the detection of Covid-19. Among the eight CNN models, ResNet-34 and Alexnet outperformed with an accuracy of 98.33 and 97.5% respectively compared to other pre-trained models. Hence the authors predicted ResNet 34 model as the potential model for the early prediction and accurate diagnosis of Covid-19 infection.

Ardakani et al.^24^ developed a CAD system based on deep learning to classify the covid-19 infection with other viral pneumonia. They used high resolution computed tomography (HRCT) images obtained from patients during the acute phase of the disease. They used transfer learning approach to optimize the ten convolutional neural networks such as Alex-Net, VGG-16, VGG-19, Squeezenet, Google net, mobile Net V2, ResNet -18, ResNet-50, ResNet-101 and Xception net for the classification of covid-19 and other pneumonia cases. Among the ten CNN used for their study, ResNet 101 and Xception network achieved the best performance accuracy of 99.51 and 99.02% respectively compared to accuracy of 86.27% obtained by the radiologists.

Therefore, In our proposed study, ResNet50, SqueezeNet, and AlexNet CNN models based on the transfer learning approach were implemented, and compared the performance of these CNN model with the machine learning classifiers such as Naïve Bayes, Bagging and REPTree. The proposed study achieved a highest accuracy of 99.1% using ResNet 50 model in comparison with other pre-trained deep learning models in classification of Covid and Non-covid images. Hence compared to the existing literatures, ResNet 50 outperformed well in the proposed study in the classification of covid-19 and normal lung CT images.

Some of the limitations of our study are discussed as follows. Firstly, the proposed research makes use of a limited dataset. However, to be more effective in clinical practice, we would require more training with a larger and a flexible dataset. Secondly, our study did not focus on the various stages of severity of infection in the lung CT images of Covid-19. All these factors could be helpful to build a more reliable model for real-life applications. The future work of our study is to develop a fully automated computer-based diagnostic system capable of diagnosing COVID-19 infection from lung CT images using the proposed method.

In conclusion, this study proposed a novel system to diagnose COVID-19 infection from lung CT images. Our study was focused on segmentation of the CT images with a k-means clustering algorithm, GLCM feature extraction, and classification based on machine learning and deep learning techniques. The Naive Bayes classifier yielded the best accuracy, precision, and sensitivity among all the three classifiers. Hence, Naive Bayes classifier was found to be the most suitable for our proposed system. The accuracy attained by the Naive Bayes classifier was 97%, with an AUC value of 0.99 from the ROC curve. Further, transfer learning approach was implemented to train three state-of-the-art CNN models such as Alex Net, ResNet50, and Squeeze Net. Out of three CNN models, ResNet50 achieved the highest accuracy of 99.1%. Hence the deep learning networks outperformed well compared to the machine learning techniques in the classification of Covid-19 images. Thus, the proposed study resulted in a more accurate classification of COVID and non-COVID subjects using chest CT images.

## Methodology

### Dataset and pre-processing

**Figure 6:**
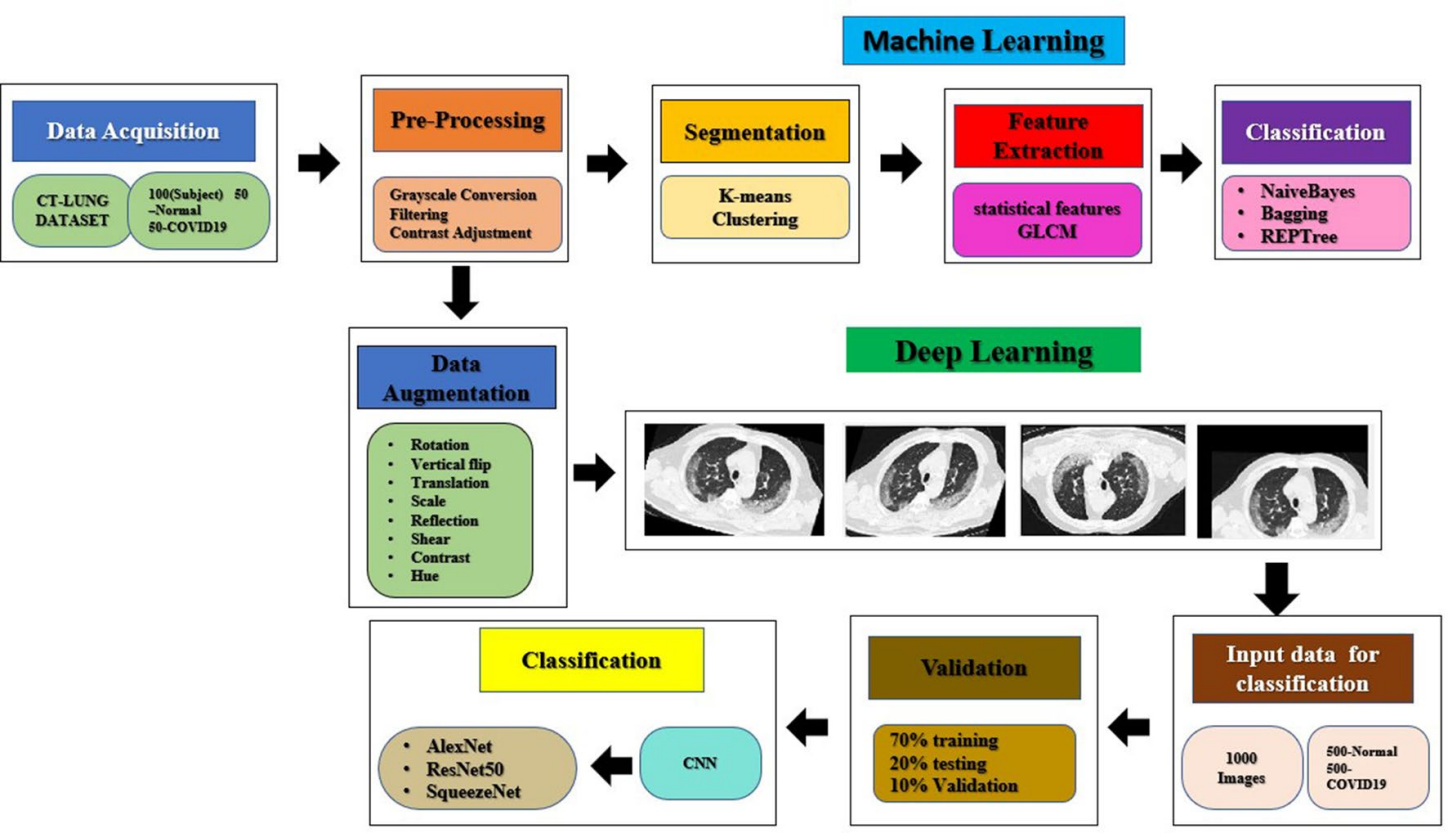
Block diagram of the proposed work.

A total of 50 lung CT images of COVID confirmed patients and 50 images of non-COVID subjects were obtained from in-patients admitted to the SRM Medical College Hospital and Research center. The confirmed patients and healthy patients' age ranged between 30 and 60 years of both male and female were included in the study. The COVID 19 patients were confirmed with moderate to severe symptoms like cough, cold, fever and difficulty in breathing. As deep learning processes require a larger dataset, data augmentation techniques such as rotation, shearing, translation, scaling, and random contrast adjustments were performed with 50 COVID images and 50 non-COVID images, thereby expanded the dataset to total 1000 images which contains 500 COVID and 500 non-COVID images. Next, pre-processing of images was performed to enhance the valuable data in the acquired images. The proposed work was approved by Institutional ethical committee with the ethical clearance number as 2844/IEC/2021. Informed consent form obtained from all the participants involved in the study. The block diagram of overall proposed study in the classification of Covid-19 and non-covid 19 images were illustrated in Fig. 6.

### *k*-means clustering algorithm

*A k*-means clustering algorithm is a data clustering algorithm which partitions the data into clusters. It is a simple iterative clustering algorithm that divided the dataset into non-overlapping clusters. The main idea behind formulating different clusters is to have unique clusters grouped with one another. The distance between the cluster centroid and data points are calculated using Euclidean distance method. *k*-means algorithm was applied to the pre-processed CT images, and the total number of clusters considered for the proposed study is 3. Cluster1 represents the infected COVID 19 region, cluster2 indicates the edges, nodules and vessels, cluster3 shows the surrounding and background region in the lung. The three resulting clusters were then analyzed and the cluster depicting the COVID-19 infection (region of interest) is selected for further process. The segmentation process was carried out in Matlab software (version R2021a) and the algorithm is summarized as follows:

(i) Enter the number of clusters *k*; Assume *k* = 3, initialize the centroid by first shuffling the dataset and then randomly selecting the *k* data points of the centroid without replacing them.
(ii) Compute the cluster centroids for each cluster
(iii) Repeat until there is no change to the centroids, this means that the data point assignments to the cluster will not change.
(iv) Calculate the sum of the squares of the distance between the data points and the centroids.
(v) Assign each data point to the closest cluster (centricity). Calculate the cluster’s centroid by averaging all the data points that belong to each cluster.

The purpose of the algorithm is to minimize objective function E, known as the squared error function, mathematically given by the following equations:

1 $$E = \mathop \sum \limits_{j = 1}^{n} \mathop \sum \limits_{k = 1}^{K} V_{{jk ||x^{j } - m_{k} ||^{2} }}$$

2 $$\frac{\partial E}{{\partial V_{jk} }} = \mathop \sum \limits_{k = 1}^{K} ||x^{j } - m_{k} ||^{2}$$

3 $$V_{{jk}} = \left\{ \begin{gathered} 1,\;\;k = ~argmin_{k} \left( {\left| {\left| {x^{j} - m_{k} } \right|} \right|} \right)^{2} \hfill \\ 0\;\;\;otherwise \hfill \\ \end{gathered} \right.$$

4 $$\frac{\partial E}{{\partial m_{k} }} = 2\mathop \sum \limits_{j = 1}^{n} V_{jk} \left( {x^{j } - m_{k} } \right) = 0$$

5 $$m_{k} = \frac{{\mathop \sum \nolimits_{j = 1}^{n} V_{jk} x^{j} }}{{\mathop \sum \nolimits_{j = 1}^{n} V_{jk} }}$$

where n is the number of data points in the k cluster, k is the number of cluster and ${m}_{k}$ is the centroid. The cluster to which ${x}^{j}$ belongs to ${V}_{jk}$ is one of the data points ${x}^{j}$. If it belongs to cluster *k*, it is 0 otherwise. This algorithm consists of two steps of minimization. In the first step, E is differentiated with respect to ${V}_{jk}$, where ${m}_{k} \mathrm{is kept}$ constant and assigned clusters are updated. In the second step, E is distinguished as ${m}_{k}$ and centroids are recalculated that depends on the sum of the square of the distance from the centroid of data point ${x}^{j}$ which is assigned to the location closest to the cluster. This iteration will continue until no further iterations are possible.

### Feature extraction

Feature extraction was carried out using Matlab (version R2021a). Six textural features were extracted such as mean, contrast, energy, entropy, homogeneity, and correlation using GLCM method. The feature extraction technique (GLCM algorithm) is based on the spatial relationship between pixels and their surroundings. Energy can be described as a measure of homogeneity in an image. A greater value of energy implies that there are more high-intensity neighbouring pixels at high frequencies. The contrast of an image is a measure of local variation in the intensity of the pixels. A significant contrast value indicates a greater variation among neighbouring pixels. Correlation computes the dependency of gray levels on their corresponding pixels in the co-occurrence matrix. Entropy is a measure of randomness of the pixels in the image. Homogeneity is a degree of similarity between the pixels. Mean is the average contribution of pixels towards the mean intensity, whereas the standard deviation measures the disparity between the pixels and the mean value. Variance is calculated by finding the square of the standard deviation^25^.

6 $${\text{Contrast}} = \mathop \sum \limits_{u,v = 0}^{N - 1} X_{uv} \left( {u - v} \right)^{2}$$

7 $${\text{Energy}} = \mathop \sum \limits_{u,v = 0}^{N - 1} \left( {X_{uv} } \right)^{2}$$

8 $${\text{Homogeneity}} = \mathop \sum \limits_{u,v = 0}^{N - 1} \frac{{X_{uv} }}{{1 + \left( {u - v} \right)^{2} }}$$

9 $${\text{Entropy}} = \mathop \sum \limits_{u,v = 0}^{N - 1} - {\text{ln}}\left( {X_{uv} } \right)\left( {X_{uv} } \right)$$

10 $${\text{Correlation}} = \mathop \sum \limits_{u,v = 0}^{N - 1} X_{uv} \frac{{\left( {u - \mu } \right)\left( {v - \mu } \right)}}{{\sigma^{2} }}$$

11 $${\text{Mean}} = \mu = \mathop \sum \limits_{u,v = 0}^{N - 1} u(X_{uv)}$$

### Classification based on machine learning techniques

Classification is a predictive modeling technique wherein a class label is predicted based on categories of the input data subjected. In the proposed study, machine learning algorithms are implemented to classify the dataset into two classes (binary classification): COVID-19 and non-COVID-19 images. The extracted features from the segmented images are used as the input attributes to facilitate the classification process. This study evaluates the performance of three classifiers, namely Naïve Bayes, Bagging, and REPTree implemented in WEKA (version 3.8).

### Naïve Bayes classifier

The Naïve Bayes classifier is a probability-based classifier that works on the principle of the Bayes theorem. It is based on conditional probability and the assumption that the attributes are independent with each other. Although this assumption is not valid for practical applications, the performance of this classifier is still on par with more complex classifiers. The Eq. (12) given below represents the Naïve Bayes classifier prediction model:

12 $$C = \arg \max_{C} P\left( C \right)\mathop \prod \limits_{i = 1}^{n} P(x_{i} | C)$$

where C = Class variable; *x*_*i*_ = Parameters/features; n = Number of features.

Naïve Bayes classifiers are simple models with excellent performance. The performance of the models may be tweaked according to individual preferences based on the application. The grid search, random search, and sequential model-based optimization (SMBO) can be implemented for hyperparameter optimization^26–28^. The hyperparameter used in Naïve Bayes classifier is alpha which is set as α = 1 and act as smoothing parameter, the batch size is set to 100. This alpha parameter improves the performance of the classifier in classification of Normal and covid-19 patients.

### Bagging classifier

The bagging classifier falls under the meta selection. Bagging classifier, also known as bootstrap aggregation, is an ensemble technique used to minimize the variance of the forecast classifier. Multiple models are trained using the same learning algorithm or classifier by using the bagging method^29^. It takes separate samples of the training dataset for classification, with the precise result; the individual classifiers are combined and most voted class is predicted as the output. This method also produces more accurate findings, as it requires a smaller number of records and allows for more robust statistical inference. Bootstrapping, building Classifiers, and aggregation are involved in the bagging classifier.

In Bootstrapping, sampling is performed on the original dataset with random substitutions to form a new dataset. Each sample is different from the original dataset, but the distribution and variance are similar. In Building classifiers, a classifier is created for each of the small datasets retrieved in the previous step. Generally, the same type of classifier is created for all records. Aggregations and each classifier effect are combined to give the final classifier output^30^. Bagging use REP Tree as a base classifier. This is a voting method; it chooses classes based on the maximum number of the sub-roots split from the main root. The batch size is set to 100 and the number of iterations for each root is 10.

### REPTree classifier

RepTree uses regression tree logic to build multiple trees with different iterations. Then the best one is selected from all the generated trees which is counted as a representative. Basically, the reduced error pruning tree (REPT) is a fast-learning decision tree that constructs a decision tree based on the information gain or variance. The REP Tree is a prompt decision tree learner that uses information gained as a split criterion to build a decision/ regression tree and to obtain error-free pruning^31^. REP Tree creates classes based on the number of roots having the maximum gain. The batch size is set to 100 with maximum tree depth as − 1. Minimum proportion of variance is given as 0.001 and Minimum total weight of instance in root is 2.

### Deep learning classification

The ImageNet Large Scale Visualisation Challenge (ILSRC) is an annual challenge to develop better machine learning and computer vision techniques. It involves the ImageNet dataset that contains thousands of annotated photographs. The goal of the challenge was to develop the classification models for image classification, object detection and recognition^32^. The trained models from the challenge are available to be used for other applications. Transfer learning was used to train three state-of-the-art CNN models. Transfer learning is the ability of deep learning networks to identify and use features such as textures and boundaries learned in earlier classification problems and exploit them for application in a new task^33^. Fine-tuning of a developed network is much easier and faster than training from scratch. In the proposed study, the AlexNet, ResNet50, and SqueezeNet architectures were used to classify COVID and non-COVID images.

### AlexNet

Alex Net was the winner of Image net large scale Visual recognition challenge (ILSRC) conducted in the year 2012, and was a revolutionary step in the advancement of deep learning. The AlexNet architecture consists of 8 deep layers, out of which 5 are convolutional layers combined with max-pooling, and the remaining 3 are fully connected layers (Fig. S1). The activation function of all the layers is the ReLU function. Prior to the development of AlexNet, most classification models used tanh activation function, leading to gradient vanishing. This was fixed by using the ReLu activation function given by *ReLU*(*x*) = *max*(*x*,*0*)*.* The gradient function is equated to one if the input is greater than 0. This activation function increased the speed of convergence of the learning task, contributing to accelerated performance.

The input images given to the Alex Net architecture are of size 227 × 227 × 3. Hence, the augmented dataset was resized before the training process. The detailed architecture of Alex net for the classification of COVID and Normal was depicted in Fig. S1. The first layer is a convolutional layer consisting of 96 kernels of stride 4 and a size of 11 × 11. The next layer is a max-pooling layer of kernel size 3 × 3 and stride 2. It is followed by the second convolutional layer with a padding of 2 and a stride of 1. This layer consists of 256 kernels of size 5 × 5. This is again followed by a max- pooling layer of size 3 × 3 and stride 2. The two layers succeeding this max-pooling layer are convolutional layers with a kernel size of 3 × 3 and a stride of 1. However, the third and fourth convolutional layers have 384 filters each, while the fifth convolutional layer has 256 filters. Following the fifth convolutional layer is an overlapping max-pooling layer of the size 3 × 3 and stride 2.

The next layer in this architecture is a dropout layer. Dropout refers to when some of the layers have "dropped out' or have not been trained during each iteration. The ratio of dropout may be fixed as well. This practice was employed to fix the problem of overfitting that is generally seen with deep learning networks. The first fully-connected layer then follows this dropout layer with the same ReLU activation as all the previous layers. Then, there is another dropout layer, wherein, the dropout rate of both the layers is fixed at 0.5. The last layer of the architecture is also fully connected, but its activation function is soft max^34,35^.

Our proposed work involves binary classification of COVID and non-COVID CT images (2 classes). Therefore, a transfer learning approach was applied to perform binary classification. All the original model parameters were preserved except for the last three layers, which were replaced with a fully-connected layer, a softmax layer, and a classification output layer. The layers of the original model serve as the initialization and are very useful for the feature extraction process as they have already been trained with the ImageNet dataset. In the proposed work, the dataset is split into mini batches of size 20 for the training process. The model was trained with a gradient-based optimization technique, with the maximum epoch set to 10. The learning rate was 1e-5.

### ResNet50

Following the widespread recognition of AlexNet architecture, the subsequent models that were developed consisted of more layers to reduce the error rate. The vanishing gradient issue becomes more pronounced when the number of layers is increased. The residual network concept was introduced to tackle this problem. In the ResNet model, some layers are skipped and connected directly to the output, this process is known as skip connections.

The ResNet50 architecture consists of 50 deep layers with ReLU as an activation function. The input image size given to the architecture is 227 × 227 × 3. There are five convolution blocks followed fully connected layer and soft max activation function. In convolution block 1, the first layer is a convolutional layer with 64 kernels of size 7 × 7 and a stride of 2, with a max pooling layer of the same stride size. It is followed by the convolution block 2, which has three layers that are executed thrice, giving us a total of 9 layers. The first layer and second layer in block 2 contain 64 kernels each of size 1 × 1, 3 × 3, respectively, and the third layer contains 256 kernels of size 1 × 1. Convolution block 3 consists of three convolutional layers that are repeated four times, yielding 12 layers. In this block, the first and second convolutional layers have 128 kernels of size 1 × 1 and 3 × 3 respectively, and the third layer has 512 kernels of size 1 × 1. The Convolution block 4 consists of the three convolutional layers that are repeated six times, yielding 18 layers. The first layer in this block has 256 kernels of size 1 × 1, the second one has 256 kernels of size 3 × 3 and the third one has 1024 layers of size 1 × 1. In convolution block 5, the three convolutional layers are present, having 512 kernels of size 1 × 1, 512 kernels of size 3 × 3 and 1024 kernels of size 1 × 1. The three layers in block 5 are executed thrice, giving us a total of 9 layers. Next to Convolution block 5, an average pooling is performed, following which the final layer is a fully-connected layer with 1000 neurons and a SoftMax activation function^36^. The detailed architecture of Resnet 50 was depicted in Fig. S2.

### SqueezeNet

The SqueezeNet architecture is a model that can achieve the equivalent accuracy as AlexNet using only half the number of parameters^37^. This Squeeze Net is a small and compact deep learning network that is as efficient as larger models due to its intelligent architecture. Three strategies are used to achieve its performance. The first strategy adopted in Squeeze Net is by reducing the kernel size from 3 × 3 to 1 × 1. The next strategy is reducing the number of input channels to 3 × 3 filters by introducing squeeze layers. The final strategy is by delayed down sampling to yield large activation maps.

Squeeze Net is constructed using a fire module that comprises a squeeze layer that has kernels of size 1 × 1 followed by an expand layer that has a mix of 1 × 1 and 3 × 3 filters. The Squeeze Net architecture has an input image of size 227 × 227 × 3 and starts with a convolutional layer of size 3 × 3 and a stride of 2. Next, there are eight fire modules in succession Max pooling of stride two is performed after the initial convolutional layer and after fourth and eighth fire modules. After the 9th fire modules, the global Average Pooling layer and then the final fully connected layer of size 1 × 1 and 1000 neurons are connected^38^. The detailed squeeze net architecture and fire module are given in Fig. S3a and b, respectively.

Using the transfer learning approach, we replaced the last two layers of this architecture with a convolutional layer with two nodes and a final convolution layer. The batch size and other training parameters were set to the same values used in the AlexNet CNN transfer learning models.

### Validation

Image classification comprises of three stages, namely training, testing, and validation. The dataset was divided into three groups where 70% of the images were used for training, 20% for testing and the remaining 10% were reserved for the validation process. Cross-validation is a technique where the machine learning model is trained with several subsets of the available data and is then evaluated on a complementary data subset. Ten-fold cross validation technique is used in the proposed method where, the dataset is divided into ten subsets or folds. It is preferred to enhance performance of the classifier and also avoids issues such as selection bias and over-fitting.

Validation of the proposed model was implemented by considering the following parameters: true positive (TP), true negative (TN), false positive (FP), and false negative (FN). The individuals affected with COVID are considered as positive and non-COVID subject are considered as negative. False positive condition occurs when the classifier model predicts a non-COVID image as a COVID positive image. False negative condition prevails when the classifier model predicts a COVID image as non-COVID image. Based on these four parameters, confusion matrices were generated for each classifier model, and performance metrics such as accuracy, precision, and sensitivity were calculated.

13 $${\text{Accuracy}} = \frac{{\left( {TP + FN} \right)}}{{\left( {TP + FP + FN + TN} \right)}}$$

14 $${\text{Precision}} = \frac{TP}{{FP + TP}}$$

15 $${\text{Sensitivity}} = \frac{TP}{{FN + TP}}$$

### Ethics approval

All procedures performed in studies involving human participants were in accordance with the ethical standards of the institutional research committee. The study was approved by the Bioethics Committee of SRM Research Centre and Hospital with Ethics Clearance Number 2844/IEC/2021.

## Supplementary Information

Supplementary Information 1.

Supplementary Information 2.

Supplementary Information 3.

Supplementary Information 4.

Supplementary Information 5.

## Data Availability

On request from the corresponding author, the data that support the findings of this study are accessible.
