# Supplementary material for: Automated system for classification of COVID-19 infection from lung CT images based on machine learning and deep learning techniques
Source: Sci Rep. 2022 Oct 18;12:17417. doi: 10.1038/s41598-022-20804-5 (PMC9579174; doi:10.1038/s41598-022-20804-5)
Supplement: Supplementary file 1 — Supplementary Information 1. [file 41598_2022_20804_MOESM1_ESM.docx]

Fig. S1 AlexNet Architecture for classification of COVID 19 and Non COVID CT images

Fig. S2 ResNet50 Architecture for the classification of COVID -19 CT images

Fig. S3a Squeeze Net Architecture for the classification of COVID-19 CT images

Fig. S3b Fire Module of Squeeze Net Architecture.
