# Supplementary figures and images for: Automated system for classification of COVID-19 infection from lung CT images based on machine learning and deep learning techniques
Source: Sci Rep. 2022 Oct 18;12:17417. doi: 10.1038/s41598-022-20804-5 (PMC9579174; doi:10.1038/s41598-022-20804-5)

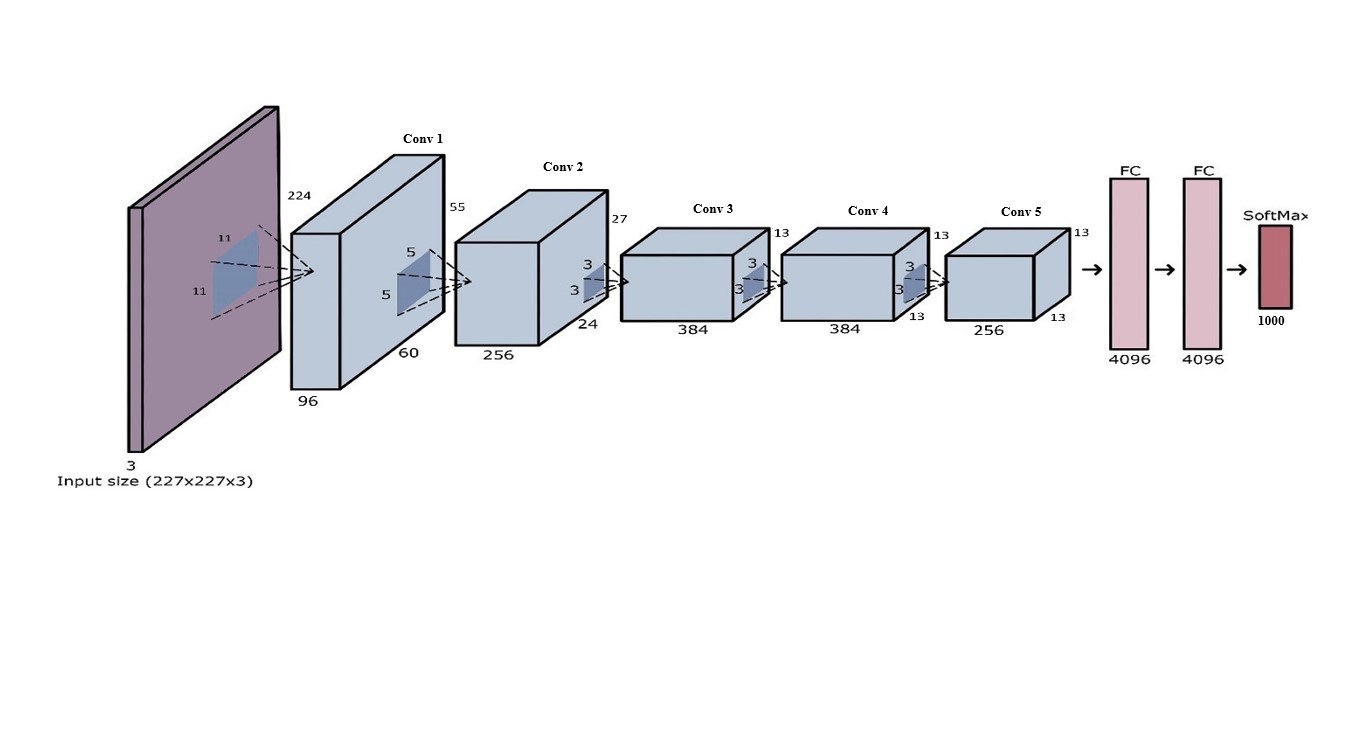

Supplement: Supplementary file 2 — Supplementary Information 2. [file 41598_2022_20804_MOESM2_ESM.jpg]

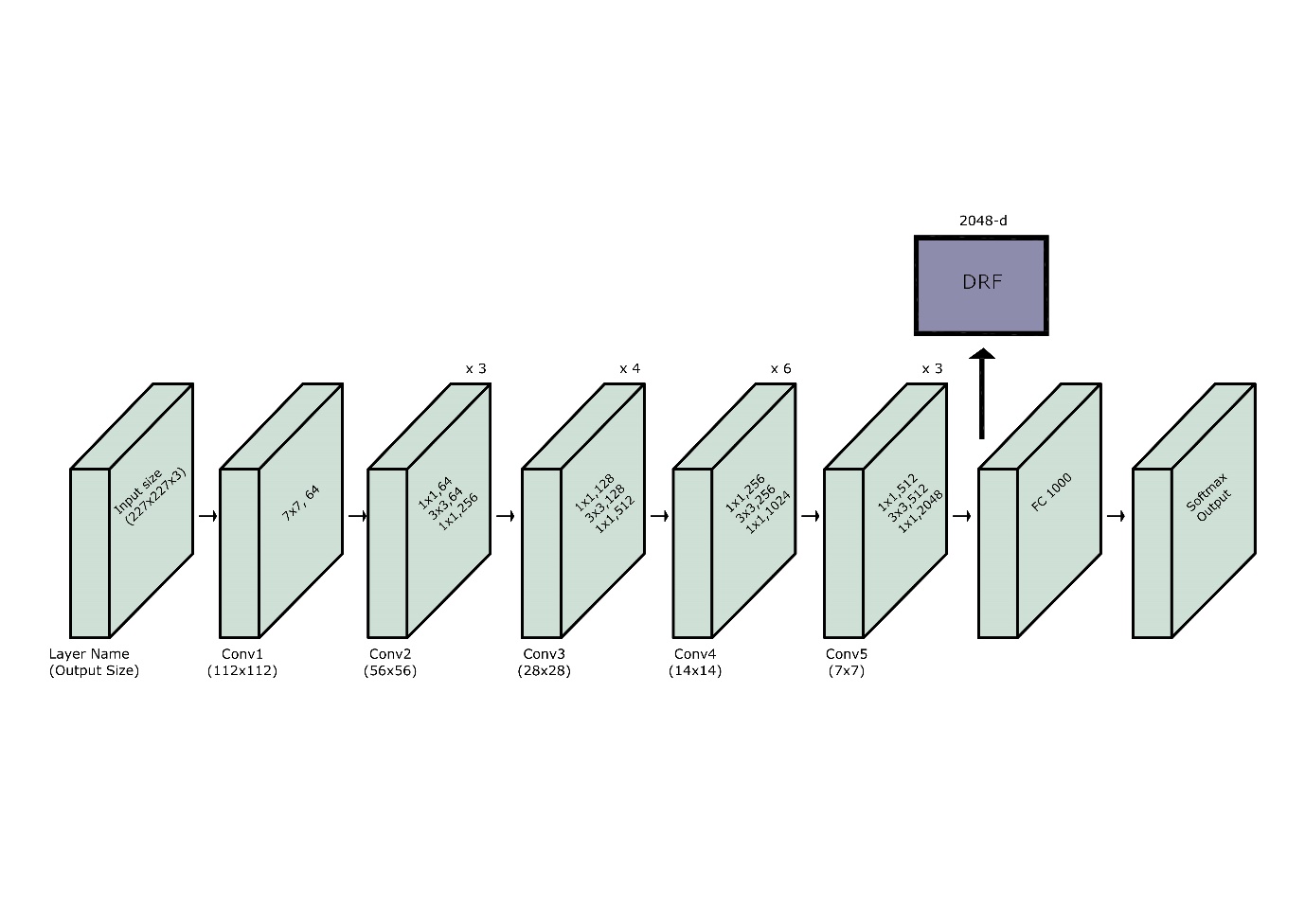

Supplement: Supplementary file 3 — Supplementary Information 3. [file 41598_2022_20804_MOESM3_ESM.jpg]

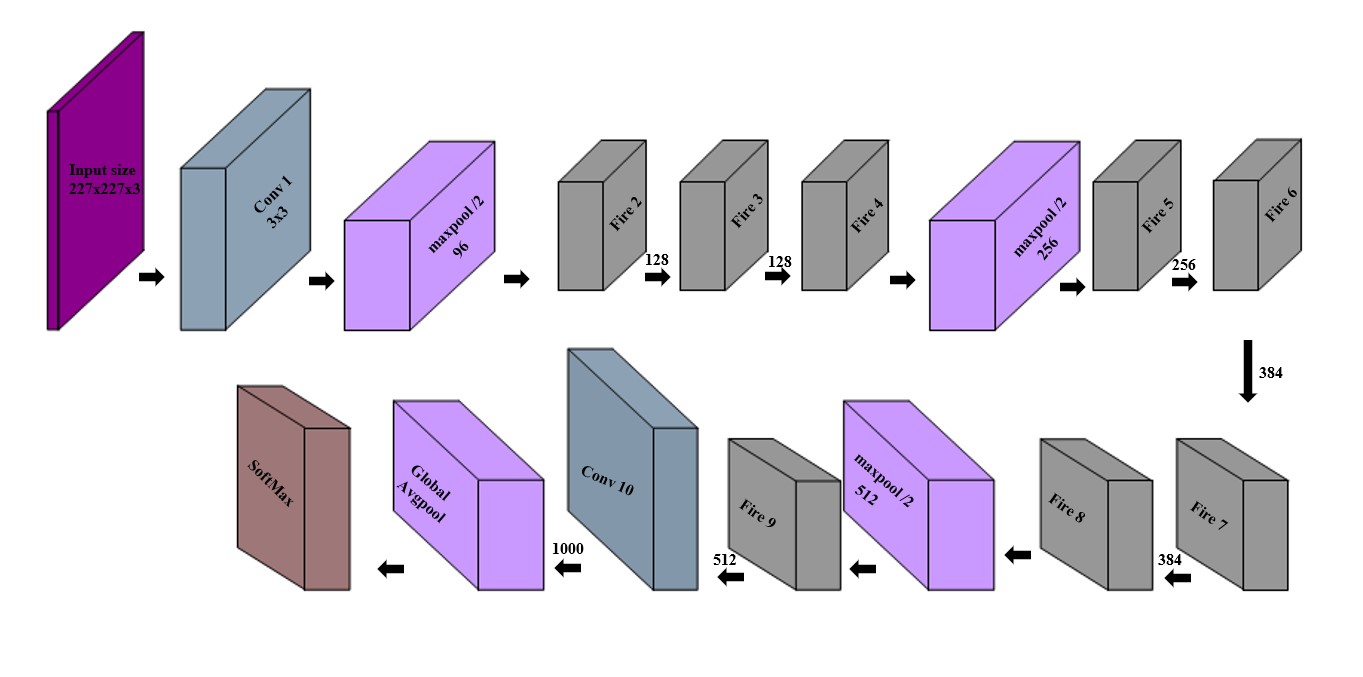

Supplement: Supplementary file 4 — Supplementary Information 4. [file 41598_2022_20804_MOESM4_ESM.jpg]

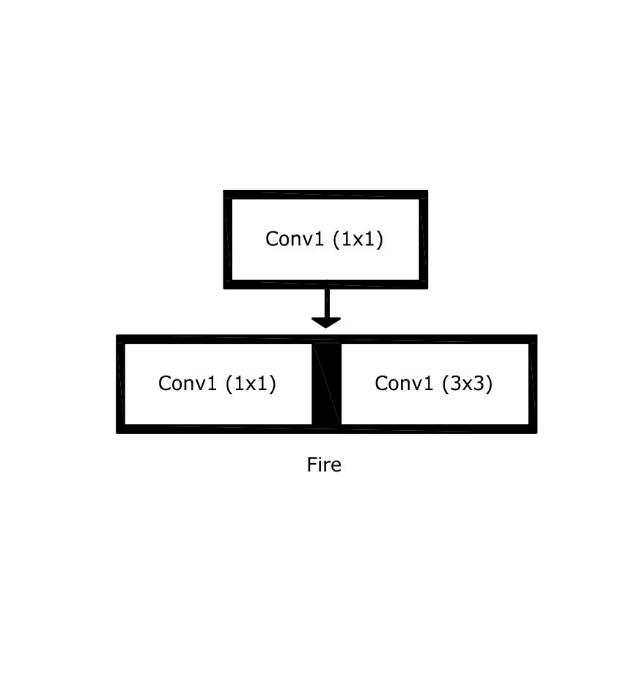

Supplement: Supplementary file 5 — Supplementary Information 5. [file 41598_2022_20804_MOESM5_ESM.jpg]
